# Thirty-Day Mortality Associated With Carbapenemase-Producing *Enterobacterales* Bloodstream Infections at a Referral Hospital in Peru, 2020–2023

**DOI:** 10.1093/ofid/ofaf729

**Published:** 2025-12-01

**Authors:** Giancarlo Pérez-Lazo, Roxana Sandoval-Ahumada, Carlos Tairo-Cerron, José Ballena-López, Fernando Soto-Febres, Steev Loyola

**Affiliations:** Posgrado de Medicina, Facultad de Ciencias de la Salud, Universidad Científica del Sur, Lima, Perú; Division of Infectious Diseases, Guillermo Almenara Irigoyen National Hospital-EsSalud, Lima, Peru; Clinical Pathology Department, Guillermo Almenara Irigoyen National Hospital-EsSalud, Lima, Peru; Division of Infectious Diseases, Guillermo Almenara Irigoyen National Hospital-EsSalud, Lima, Peru; Division of Infectious Diseases, Guillermo Almenara Irigoyen National Hospital-EsSalud, Lima, Peru; Division of Infectious Diseases, Guillermo Almenara Irigoyen National Hospital-EsSalud, Lima, Peru; Posgrado de Medicina, Facultad de Ciencias de la Salud, Universidad Científica del Sur, Lima, Perú

**Keywords:** antimicrobial resistance, bloodstream infection, carbapenem-resistant *Enterobacterales*, KPC, NDM

## Abstract

**Background:**

Bloodstream infections (BSIs) caused by carbapenemase-producing *Enterobacterales* (CPE) are associated with increased mortality rates. However, limited data exist in Latin America, particularly in regions where *bla*_NDM_ is predominant.

**Methods:**

We conducted a retrospective cohort study of adult inpatients with *Escherichia coli* or *Klebsiella pneumoniae* BSI at a Peruvian referral hospital between 2020 and 2023. Patients were classified as CPE or non-CPE based on molecular testing. The outcome was 30-day all-cause mortality. Multivariable Cox regression was used to identify independent predictors of mortality, adjusting for confounders selected via a directed acyclic graph.

**Results:**

Among 506 patients, 97 (19.2%) had CPE BSIs, predominantly *bla*_NDM_ (63.9%) and *bla*_KPC_ (36.1%). Overall, the 30-day mortality rate was 27.3%, significantly higher in the CPE group (52.6% vs 21.3%; *P* < .001). In multivariable analysis, CPE infection remained independently associated with increased mortality (adjusted hazard ratio [aHR] 1.88; 95% CI 1.19–2.96). Other predictors included age ≥60 years (aHR 1.53), septic shock (aHR 2.93), pneumonia (aHR 1.70), and immunosuppression (aHR 1.72). Among CPE subtypes, *Klebsiella pneumoniae* Carbapenemase (KPC)-producers conferred the highest mortality risk (aHR 2.64). Concordant empirical antibiotic therapy was not significantly protective after adjustment.

**Conclusions:**

CPE BSIs were independently associated with increased 30-day mortality, with KPC-producing *K pneumoniae* posing the greatest risk. Despite the predominance of New Delhi Metallo-β-Lactamase (NDM) in our setting, these findings emphasize the clinical severity associated with different carbapenemase types, and the need to tailor interventions accordingly. Strengthening molecular surveillance and ensuring timely access to effective therapies remain critical priorities in NDM-endemic regions, such as Peru.

## Teaser

This study analyzed 506 bloodstream infections caused by *Escherichia coli* or *Klebsiella pneumoniae* in a Peruvian hospital. Carbapenemase-producing *Enterobacterales*, especially *bla*_KPC_ producers, have been linked to increased 30-day mortality, underscoring the need for molecular surveillance and improved therapeutic access.

## BACKGROUND

*Escherichia coli* and *Klebsiella pneumoniae* resistant to carbapenems are classified as critically important *Enterobacterales* by the World Health Organization (WHO) due to their public health impact and the urgent need for new and effective antibiotics targeting these pathogens [1, 2]. Compared to carbapenem-susceptible strains, carbapenem-resistant *Enterobacterales* (CRE) are associated with higher attributable mortality, prolonged courses of antimicrobial therapy, extended hospital stays, and increased healthcare costs related to infection management [3–5].

The most frequent mechanism of carbapenem resistance among *Enterobacterales* is the production of carbapenemases, with *Klebsiella pneumoniae* Carbapenemase (KPC), Verona integron-encoded metallo-β-Lactamase (VIM), Imipenemase-Type Metallo-β-Lactamase (IMP), New Delhi Metallo-β-Lactamase (NDM), and Oxacillin-hydrolyzing β-Lactamase (OXA-48) being the most widely distributed and studied types globally [6]. In Latin America, the COVID-19 pandemic has coincided with an increase in infections caused by carbapenemase-producing *Enterobacterales* (CPE) [7–9], including reports of novel carbapenemases and strains co-harboring multiple resistance genes (eg, *bla*_KPC_ + *bla*_NDM_ in *K. pneumoniae*; *bla*_OXA-48–like_ + *bla*_NDM_ in *E. coli*) [9]. However, several countries in the region still face limited molecular detection capacity due to under-implementation of advanced testing methods [9].

Emerging evidence suggests that metallo-β-lactamases (MBLs) may be associated with higher mortality rates than other carbapenemase types. For example, a prospective multicenter study conducted in 19 Italian hospitals between 2018 and 2020, involving 1276 patients with gram-negative bloodstream infections (GN-BSI), reported a 30-day mortality rate of 36.4% among patients infected with MBL-producing *Enterobacterales* and 26.6% for *K. pneumoniae* harboring KPC, compared to 13.7% in patients with carbapenem-susceptible GN-BSI [5]. Conversely, data summarized by Boyd et al. indicate that mortality in severe infections due to *Enterobacterales* with *bla*_NDM_ appears to be relatively low, ranging from 13% to 55%, compared to 18%–67% for other MBLs and 41%–65% for KPC producers. These findings highlight that mortality data for NDM-producing isolates remain conflicting [10].

In Latin America, data regarding the clinical impact of CPE remain scarce. A prepandemic multicenter study conducted in 11 hospitals across 7 countries (Argentina, Colombia, Ecuador, Guatemala, Mexico, Peru, and Venezuela) reported a 28-day mortality rate of 64% among patients with CPE bloodstream infections (BSI) compared to 30% among those with non-CPE infections [11]. Unlike other South American countries where *bla*_KPC_ predominates [11], data from hospitals in Lima, Peru, suggest that *bla*_NDM_-type MBLs were more prevalent in the prepandemic period [12, 13]. Additionally, a multicenter study involving 15 Peruvian hospitals estimated an overall in-hospital mortality rate of 33.3% for GN-BSI, without specifically evaluating the impact of carbapenemase production [14].

Despite growing concern over CPE in Latin America, there is a significant gap in clinical data from Peru regarding the impact of these infections, particularly in terms of patient outcomes and the role of different carbapenemase types. While surveillance of antimicrobial resistance has expanded, no studies to date have directly compared 30-day mortality outcomes between CPE and non-CPE BSI in referral Peruvian hospitals. This lack of localized, molecularly informed outcome data hinders effective clinical management and policy development in a setting where diagnostic capacity remains limited. This study aimed to describe and compare 30-day all-cause mortality in patients with BSI caused by CPE, focusing on the two predominant carbapenemase genotypes in our setting—KPC and NDM—and to contrast outcomes with those from non-CPE BSI.

## METHODS

### Population and Study Design

We conducted a retrospective cohort study and secondary data analysis at Guillermo Almenara Irigoyen National Hospital (HNGAI), a tertiary center affiliated with the Peruvian Social Security System (EsSalud) in Lima, Peru. The HNGAI is a national referral center with 1116 inpatient beds and an average of 23 113 discharges per year. It hosts specialized units for critical care, transplantation, and complex surgeries. For this analysis, the study period spanned from 1 January 2020 to 31 December 2023. This report follows the Strengthening the Reporting of Observational Studies in Epidemiology (STROBE) guidelines.

### Study Population

The study population included hospitalized adult (≥18 years old) patients diagnosed with BSI due to *E. coli* or *K. pneumoniae*, based on the Centers for Disease Control and Prevention/National Healthcare Safety Network [CDC/NHSN] surveillance criteria for laboratory-confirmed infection [15]. Only the first bacteremia episode per patient was considered.

Exclusion criteria were as follows: polymicrobial BSI, identification of carbapenemase genes performed at external laboratories, co-detection of two or more carbapenemase genes, rehospitalization for a bacteremia episode with the same resistance profile, transfer to non–EsSalud facilities before completion of follow-up or outcome assessment, incomplete outcome data, and isolates harboring *bla*_OXA-48_ due to their low frequency, which precluded meaningful subgroup analyses.

Patients with CPE constituted the exposed group, while those without CPE formed the unexposed group. The non-CPE group included patients with bacteremia due to *E. coli* or *K. pneumoniae* isolates in which no carbapenemase genes were detected, regardless of their carbapenem susceptibility profile. CPE cases were further categorized according to carbapenemase genotype into KPC- or NDM-producing CRE.

### Sample Size

A minimum of 330 patients (98 exposed and 232 unexposed) was estimated to detect a difference in 30-day mortality, assuming rates of 26.6% in the exposed and 13.7% in the unexposed group, with a 2.38 ratio of unexposed to exposed patients [5 ]. All eligible patients during the study period were included. The final sample consisted of 97 CPE cases and 409 non-CPE cases. A post hoc power analysis using OpenEpi confirmed a statistical power >90% with continuity correction [16].

### Outcome Definition and Follow-up

The index date (day 0) was defined as the date of the blood culture sampling. Patients were followed for 30 days from the index date. The primary outcome was all-cause 30-day mortality, defined as death occurring within 30 days of the index date [17]. Mortality status at 30 days was determined using the EsSalud electronic health record system, which integrates inpatient and outpatient encounters and mortality registries across the national network. This allowed us to capture deaths that occurred after hospital discharge within the 30-day follow-up window.

### Definitions

Immunosuppression: chemotherapy in the past 6 months, solid organ or hematopoietic stem cell transplant, chronic corticosteroid therapy, or neutropenia (<200 cells/mL) on the day of bacteremia.

CPE colonization: detection of a carbapenemase gene in a rectal swab performed within 6 months preceding the bacteremia episode, as part of routine surveillance in patients with known contact with CPE carriers or during Intensive Care Unit (ICU) admission [18].

Persistent bacteremia: repeated isolation of the same pathogen from ≥2 blood cultures obtained at different times despite antibiotic therapy during hospitalization [19].

Discordant antimicrobial therapy: failure to prescribe an active antimicrobial agent to which the organism was susceptible or administration of an agent to which the organism was resistant [20].

Adequate source control: removal of an invasive device or drainage of abscess/collection within 72 hours of infection onset [21].

Combination therapy was defined as the concurrent administration of ≥2 systemic antimicrobials directed at *Enterobacterales*; classification was independent of Clinical and Laboratory Standards Institute (CLSI) interpretive categories (eg, colistin categorized as intermediate). Monotherapy was defined as the use of one such agent [22].

### Data Collection and Quality Assurance

Three trained infectious disease physicians extracted data from electronic medical records using a standardized clinical report form (CRF) in REDCap. CRF included demographic data, clinical characteristics, bacteremia episode details, treatment variables, antimicrobial susceptibility, and outcomes. All data were de-identified. A data manager performed routine quality control by auditing full CRFs for completeness and accuracy. Interobserver agreement was assessed on 10% of randomly selected extracted records for selected variables (discordant antimicrobial therapy, infection source, and mortality outcome).

### Microbiological Methods

Blood isolates were processed at the HNGAI Microbiology Laboratory following standard procedures. Initial organism identification and antimicrobial susceptibility testing were performed with the VITEK-2 GN platform (bioMérieux, Marcy l’Etoile, France).

Carbapenemase production was confirmed by using a stepwise algorithm: screening for ertapenem nonsusceptibility (minimum inhibitory concentration [MIC] >0.5 µg/mL); phenotypic confirmation using the modified carbapenem inactivation method (mCIM), disk synergy testing with boronic acid or EDTA, and lateral flow testing (RESIST-5 O.K.N.V., Coris Bio-Concept); and molecular confirmation using the Xpert Carba-R assay (Cepheid) to detect *bla*_KPC_ and *bla*_NDM_ genes.

Colistin resistance was assessed by the ColiSpot agar method. MIC values were interpreted according to the CLSI breakpoints applicable for each study year [23].

### Statistical Analysis

Descriptive statistics were used to summarize baseline characteristics, including demographics (eg, age and sex), comorbidities, and clinical parameters, such as the Charlson comorbidity index and Pitt bacteremia score. Continuous variables were expressed as medians with interquartile ranges (IQR), and categorical variables as frequencies and percentages. Survival analysis was conducted using Kaplan–Meier curves and the log-rank test to compare 30-day mortality between CPE and non-CPE groups, and among carbapenemase genotypes (KPC and NDM) versus non-CPE. Independent predictors of 30-day all-cause mortality were identified using a multivariable Cox proportional hazard model. Covariates included age, septic shock at bacteremia onset, pneumonia as the infection source, immunosuppression, and concordant empirical therapy, which were selected a priori using a directed acyclic graph (Supplementary Figure 1). Variables representing early severity or level of care (eg, ICU admission at onset and Pitt bacteremia score) were conceptualized as postexposure mediators and therefore excluded from the main adjustment set to avoid overadjustment. No collinearity was detected, and proportional hazards assumptions were met (global test: χ² = 6.32; *P* = .388) (Supplementary Table 1).

In addition, a sensitivity analysis was conducted excluding patients with COVID-19 as a comorbidity, to evaluate the potential confounding effect of pandemic-related mortality.

All analyses were performed using Stata version 18.0 (StataCorp, College Station, Texas, USA). Statistical significance was set at a 2-sided *P* value ≤ .05. Graphs were created using GraphPad Prism v10.

### Ethics

This study was conducted in accordance with the Declaration of Helsinki and the national and institutional ethical standards. Ethical approval was obtained from the Ethics Committee of Universidad Científica del Sur (approval code: POS-50-2024-00696) and the Institutional Review Board of the Hospital Nacional Guillermo Almenara Irigoyen (approval code 102-2024).

## RESULTS

### Study Population

**Figure 1.**
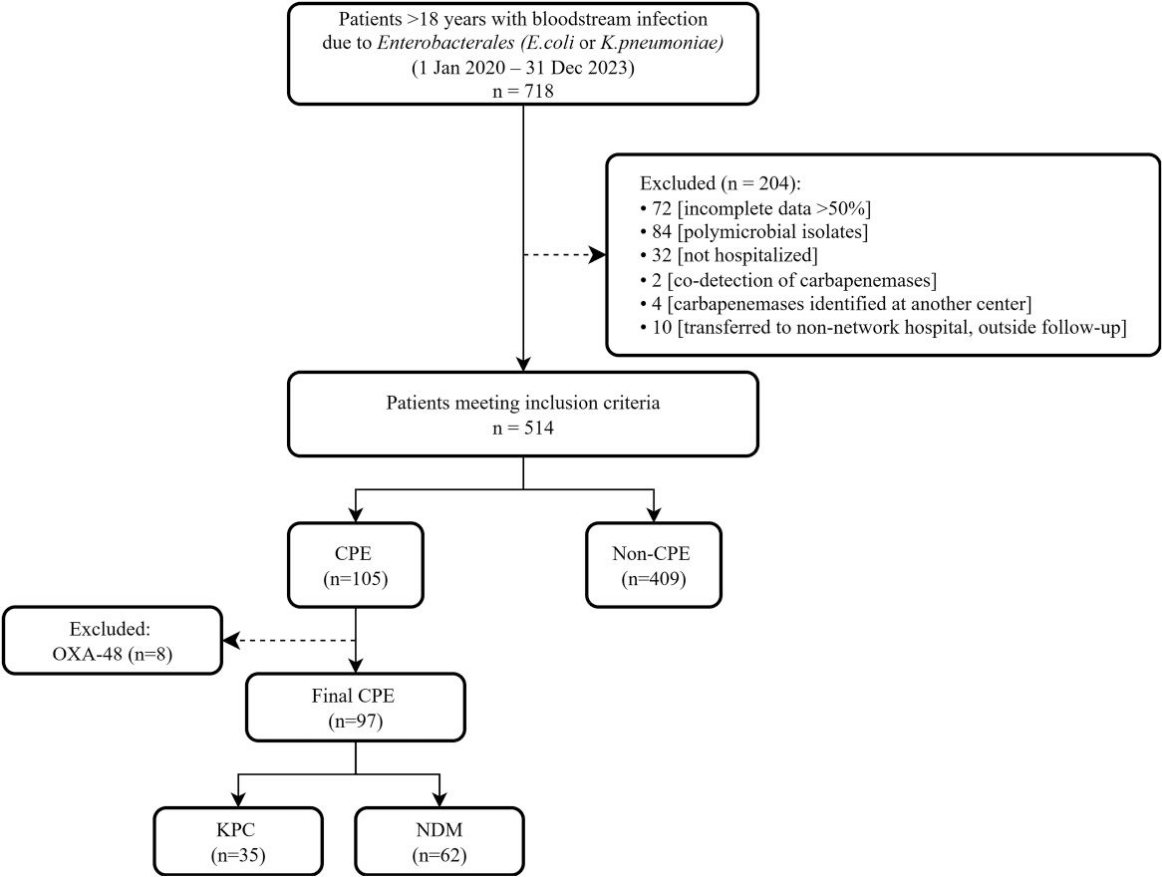
Study participant flowchart. CPE, carbapenemase-producing *Enterobacterales.*

Between 1 January 2020 and 31 December 2023, a total of 718 nonduplicate blood culture isolates of *E. coli* and *K. pneumoniae* were identified. Of these, 514 met the eligibility criteria and were included in the final analysis (Figure 1). Ultimately, 97 patients (19.2%) had CPE BSI, whereas 409 (80.8%) had non-CPE infections. Among CPE BSI cases, 63.9% isolates (*n* = 62) harbored *bla*_NDM_ and 36.1% (*n* = 35) harbored *bla*_KPC_. No isolates were positive for *bla*_IMP_ or *bla*_VIM_.

The overall median age of patients was 64 years (IQR: 49–75 years), and 58.1% were male. The most common sources of bacteremia were the urinary tract (31.4%), intra-abdominal sites (25.5%), and lower respiratory tract (17.8%). *Klebsiella pneumoniae* accounted for 95.9% of CPE isolates, whereas *E. coli* was predominant in the non-CPE group (65.8%).

### Clinical Characteristics of CPE vs Non-CPE Infections

Patients with CPE cases were more likely to be admitted to ICU at the day of bacteremia onset, present with septic shock, and have a history of recent surgery or COVID-19. A total of 29.8% of bacteremia episodes occurred in immunocompromised patients, with a similar distribution across groups. The Pitt bacteremia score was significantly higher in the CPE group (median 5 vs 2, *P* < .001). Rectal colonization with CPE was documented in only 2.7% of patients. Additional clinical and microbiological characteristics are presented in Table 1 and Supplementary Tables 2 and 3. Global inter-observer agreement for data abstraction was high (κ = 0.994), indicating minimal variability between observers.

**Table 1.** Distribution of Bloodstream Infection Episodes by Group

Variable	CPE (*n* = 97)	Non-CPE (*n* = 409)	*P* Value
Sex			
Male, *n* (%)	65 (67.0)	229 (56.0)	.048
Age, median (IQR)	57 (44–68)	66 (52–76)	<.001
Hospital service at onset, *n* (%)			
ICU	53 (54.7)	49 (12.0)	<.001
General medical ward	23 (23.7)	122 (29.8)	.231
Surgical ward	11 (11.3)	37 (9.1)	.488
Emergency department	10 (10.3)	201 (49.1)	<.001
Comorbidities, *n* (%)			
Diabetes mellitus	20 (20.6)	100 (24.5)	.425
Chronic kidney disease	17 (17.5)	106 (25.9)	.083
Postsurgical status	35 (36.1)	90 (22.0)	.004
COVID-19	19 (19.6)	27 (6.6)	<.001
Immunosuppression, *n* (%)			
Any immunosuppressed condition	30 (30.9)	121 (29.6)	.795
Chemotherapy in past 6 m	9 (9.3)	58 (14.2)	.200
Solid organ transplant	7 (7.2)	13 (3.2)	.081
Bone marrow transplant in past year	1 (1.0)	2 (0.5)	.473
Chronic corticosteroid therapy	6 (6.2)	26 (6.4)	.950
Neutropenia (<200 cells/µL on day 1)	7 (7.2)	42 (10.3)	.361
Charlson comorbidity index, median (IQR)	4 (2–5)	5 (3–6)	<.001
Source of bacteremia, *n* (%)			
Urinary tract	14 (14.4)	145 (35.5)	<.001
Catheter-related	15 (15.5)	47 (11.5)	.283
Pneumonia	38 (39.2)	52 (12.7)	<.001
Intra-abdominal	15 (15.5)	114 (27.9)	.012
Skin and soft tissue	7 (7.2)	19 (4.7)	.302
Pitt bacteremia score on day 1, median (IQR)	5 (3–6)	2 (0–3)	<.001
Septic shock at onset, *n* (%)	45 (46.4)	109 (26.7)	<.001
ICU admission on day 1, *n* (%)	62 (63.9)	57 (14.0)	<.001
Pathogen, *n* (%)			<.001
* Klebsiella pneumoniae*	93 (95.9)	140 (34.2)	
* Escherichia coli*	4 (4.1)	269 (65.8)	

Abbreviations: CPE, carbapenemase-producing *Enterobacterales*; ICU, intensive care unit; IQR, interquartile range.

### Primary Outcome

The overall 30-day all-cause mortality rate was 27.3%, significantly higher in the CPE group compared to the non-CPE group (52.6% vs 21.3%, 95% CI 0.15–0.39; *P* < .001). Among the CPE subgroups, mortality was 65.7% for patients with KPC-producing *K. pneumoniae* and 45.2% for those with NDM-producing strains. Compared to non-CPE cases, KPC infections were associated with a markedly increased mortality risk (95% CI 3.39–14.82; *P* < .001), as were NDM-producing isolates (95% CI 1.75–5.30; *P* < .001).

**Figure 2.**
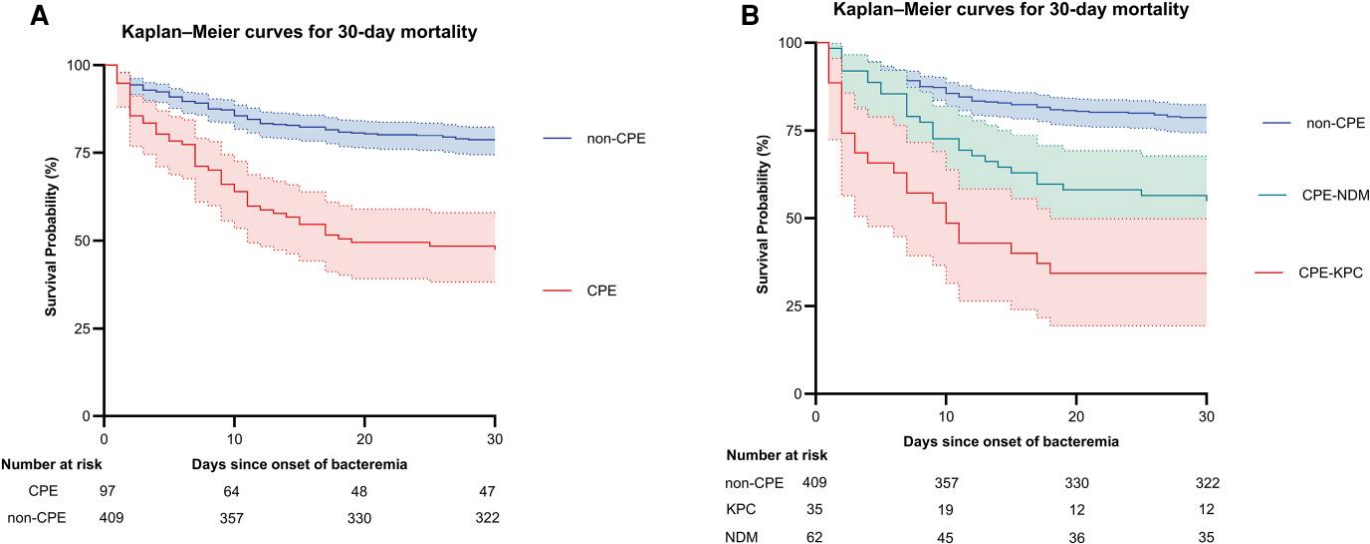
Kaplan–Meier survival estimates for 30-day all-cause mortality in patients with *Enterobacterales* bacteremia. *A*, Survival probability of patients with carbapenemase-producing *Enterobacterales* (CPE, red line) versus non-carbapenemase-producing *Enterobacterales* (non-CPE, blue line). The shaded areas represent 95% confidence intervals. Survival was significantly lower in the CPE group (*P* < .001; log-rank test). *B*, Survival estimates stratified by CPE subtype: KPC-producing (red line), NDM-producing (green line), and non-CPE (blue line). Statistically significant differences were observed between groups (*P* < .001; log-rank test).

The 30-day mortality incidence rate was 2.83 per 100 person-days in the CPE group, and 0.84 per 100 person-days in the non-CPE group. Kaplan–Meier analysis demonstrated a significantly reduced survival probability in CPE BSI (*P* < .001, Figure 2*A*). Subgroup analyses further revealed differences between the KPC and non-CPE, NDM and non-CPE, and KPC and NDM groups (*P* < .001, *P* < .001, and *P* = .015, respectively; Figure 2*B*).

Notably, among species-specific infections, the 30-day mortality rate was 50% in *E. coli* with NDM (2/4), 65.7% in KPC-producing *K. pneumoniae* (23/35), and 44.8% in NDM-producing *K. pneumoniae* (26/58).

### Factors Associated With Mortality

A comparison of survivors and nonsurvivors revealed significant differences in various clinical characteristics (Table 2). Nonsurvivors had a higher prevalence of pneumonia as the source of bacteremia and septic shock at onset, and were more likely to be immunocompromised or require ICU admission. They also received concordant empirical antibiotics and source control measures less frequently. Median Pitt bacteremia and Charlson comorbidity scores were also significantly higher among nonsurvivors.

**Table 2.** Comparison Between 30-Day Survivors and Nonsurvivors of *Enterobacterales* Bacteremia

Variable	Survivors (*n* = 368)	Nonsurvivors (*n* = 138)	*P* Value
Age, median (IQR)	64 (49–73)	66.5 (53–79)	.038
Male sex, *n* (%)	211 (57.3)	83 (60.1)	.569
Hospital ward, *n* (%)			
Emergency department	155 (42.1)	56 (40.6)	.754
Surgical ward	38 (10.3)	10 (7.2)	.292
Medical ward	109 (29.6)	36 (26.1)	.434
ICU	66 (18.0)	36 (26.1)	.042
Comorbidities, *n* (%)			
Diabetes mellitus	87 (23.6)	33 (23.9)	.949
Chronic kidney disease	81 (22.0)	42 (30.4)	.049
Postsurgical status	88 (23.9)	37 (26.8)	.501
COVID-19	34 (9.2)	12 (8.7)	.850
Immunosuppressed status	102 (27.7)	49 (35.5)	.088
Chemotherapy (last 6 m)	46 (12.5)	21 (15.2)	.422
Solid organ transplant	15 (4.1)	5 (3.6)	.816
Bone marrow transplant (last year)	3 (0.8)	0 (0)	.566
Chronic corticosteroid therapy	22 (5.9)	10 (7.3)	.602
Neutropenia <200 cells/μL on day 1	34 (9.2)	15 (10.8)	.581
Charlson comorbidity index, median (IQR)	4 (2–6)	5 (4–8)	<.001
Pitt bacteremia score, median (IQR)	2 (0–3)	4 (3–6)	<.001
Source of bacteremia, *n* (%)			
Urinary tract	125 (33.9)	34 (24.6)	.044
Catheter-related	49 (13.3)	13 (9.4)	.234
Pneumonia	46 (12.5)	44 (31.9)	<.001
Intra-abdominal	102 (27.7)	27 (19.6)	.061
Skin and soft tissue	20 (5.4)	6 (4.4)	.622
Septic shock at onset, *n* (%)	79 (21.5)	75 (54.4)	<.001
Concordant empirical antibiotic therapy, *n* (%)	267/365 (73.2)	74/138 (53.6)	<.001
Adequate source control, *n* (%)	132/206 (64.1)	7/60 (11.7)	<.001
CPE pathogen, *n* (%)	46 (12.5)	51 (36.9)	<.001

Abbreviations: CPE, carbapenemase-producing *Enterobacterales*; ICU, intensive care unit; IQR, interquartile range.

In multivariable Cox regression analysis (Table 3), independent predictors of increased 30-day mortality included age ≥60 years (adjusted hazard ratio [aHR]: 1.53; 95% CI 1.06–2.19), septic shock at onset (aHR 2.93; 95% CI 2.06–4.17), pneumonia as the infection source (aHR 1.70; 95% CI 1.15–2.52), and immunosuppression (aHR 1.72; 95% CI 1.20–2.46). CPE infection remained independently associated with increased mortality compared to non-CPE (aHR 1.88; 95% CI 1.19–2.96; *P* = .006). While concordant empirical antibiotic therapy was protective in the bivariate analysis (HR: 0.51; 95% CI 0.36–0.71), this association did not remain significant in the adjusted model (aHR 0.72; 95% CI 0.47–1.07; *P* = .105).

**Table 3.** Bivariate and Multivariable Analysis of 30-Day Mortality in Patients With *Enterobacterales* Bacteremia

Variable	Bivariate Analysis			Multivariable Analysis		
	HR	95% CI	*P* Value	aHR	95% CI	*P* Value
Age ≥60 y (vs <60)	1.25	0.88–1.77	.220	1.53	1.06–2.19	.023
Septic shock at onset	3.45	2.46–4.82	<.001	2.93	2.06–4.17	<.001
Pneumonia (vs nonpulmonary)	2.52	1.76–3.60	<.001	1.70	1.15–2.52	.008
Concordant empirical therapy	0.51	0.36–0.71	<.001	0.72	0.47–1.07	.105
Immunosuppressed status	1.38	0.97–1.95	.071	1.72	1.20–2.46	.003
CPE pathogen (vs non-CPE)	3.01	2.13–4.26	<.001	1.88	1.19–2.96	.006

Abbreviations: aHR, adjusted hazard ratio; CI, confidence interval; CPE, carbapenemase-producing *Enterobacterales*; HR, hazard ratio. Multivariable analysis adjusted for all variables listed in the table.

Exploratory analyses (Supplementary Table 4) suggested that KPC-producing strains were associated with the highest mortality risk (aHR 2.64; 95% CI 1.52–4.59; *P* = .001), whereas NDM-producing isolates showed a trend toward lower risk compared to KPC (aHR 0.57; 95% CI 0.31–1.01; *P* = .055).

### Sensitivity Analysis

To assess the potential confounding effect of COVID-19, we conducted a sensitivity analysis excluding patients with COVID-19 as a comorbidity (*n* = 46). The association between CPE BSI and 30-day all-cause mortality remained significant in this restricted cohort (aHR 2.27; 95% CI 1.39–3.71; *P* = .001). The predictors of mortality were consistent with those of the main model, including age ≥60 years, pneumonia, septic shock, and immunosuppression, whereas concordant empirical therapy was not significantly associated with survival (Supplementary Table 5 and Supplementary Figure 2).

As *K. pneumoniae* was the predominant species among CPE isolates, whereas *E. coli* predominated among non-CPE isolates, we also performed a subgroup analysis restricted to *K. pneumoniae* BSI (*n* = 233) to explore potential species-related confounding. In this subgroup, patients with CPE isolates had higher clinical severity and poorer initial management, including higher rates of septic shock (46.2% vs 29.3%), ICU admission (65.6% vs 20.9%), and discordant empirical therapy (77.4% vs 24.3%) (Supplementary Table 6). The adjusted association between CPE infection and 30-day mortality was attenuated and no longer statistically significant (aHR 1.44; 95% CI 0.84–2.47; *P* = .189), likely reflecting the greater baseline severity and smaller sample size in this subgroup (Supplementary Table 6 and Supplementary Figure 2).

### Antibiotic Treatment

Of the 503 patients who received empirical antibiotic therapy, 67.8% (*n* = 341) received concordant treatment. Carbapenems were the most frequently used antibiotics in both CPE (56.8%) and non-CPE (43.9%) groups. Among CPE cases, 17.0% (*n* = 21) received colistin as part of the empirical regimen, while β-lactam/β-lactamase inhibitor combinations (eg, piperacillin-tazobactam and ampicillin-sulbactam) were used in 30% (*n* = 127) of non-CPE cases. Empirical combination therapy was more frequently used in the CPE group (18.6%, 18/97).

Definitive therapy was administered to 462 of 506 patients (91.3%). Significant differences were observed between groups in the use of combination therapy versus monotherapy: 86.8% of patients with CPE received combination therapy compared to 13.3% in the non-CPE group (*P* < .001), while monotherapy was used in 2.1% and 97.9% of patients, respectively (*P* < .001). In the CPE group, 90% (*n* = 72) of patients received definitive colistin-based regimens. The most frequent combination was colistin and tigecycline (57.4%, 39/68).

Details of definitive antibiotic regimens and their associated 30-day mortality outcomes in the CPE group are provided in Supplementary Table 7. For colistin-based combination regimens, the overall 30-day mortality was 44.1%, with a wide variability across specific combinations, ranging from 0% to 100%.

### Antimicrobial Susceptibility Profile

Profiles are summarized in Supplementary Table 8. In the CPE group, amikacin (92.8%) and tigecycline (56.7%) showed the highest in vitro activity, while 93.8% of isolates were categorized as intermediate to colistin. Notably, no ceftazidime/avibactam-resistant isolates were identified among KPC-producing *Enterobacterales*. A high level of resistance to carbapenems was observed in the CPE group, with 97.9% (95/97) of the isolates having meropenem MICs ≥16 µg/mL. In contrast, the non-CPE group demonstrated high susceptibility to carbapenems (ertapenem, 99.1%; meropenem, 99.8%; imipenem, 99.6%) and amikacin (95.1%). Extended-spectrum β-lactamases (ESBLs) were identified in 58.7% (240/409) of non-CPE isolates.

## DISCUSSION

In this study, patients with CPE BSI had significantly higher 30-day all-cause mortality compared to those with non-CPE infections (52.6% vs 21.3%). This finding aligns with a meta-analysis by Budhram et al., which reported a 25% absolute increase in in-hospital mortality associated with CPE bacteremia (95% CI 17%–32%) [24]. Similarly, Villegas et al. reported higher mortality in patients with CPE BSI compared to those with non-CPE BSI (64% vs 30%) in a multicenter Latin American cohort, predominantly involving isolates harboring *bla*_KPC_ (83%), *bla*_VIM_ (9%), and *bla*_NDM_ (8%) [11].

In contrast, our cohort was predominantly composed of *bla*_NDM_-producing isolates, in line with recent surveillance data from the ATLAS 2020–2022 program in Latin America [25]. National data from Peru (2023–2024) support this trend, showing that over 90% of CRE isolates from 10 referral hospitals were *E. coli* or *K. pneumoniae*, with *bla*_NDM_ identified in 59.9%, *bla*_KPC_ in 25.3%, *bla*_OXA-48_ in 8.0%, and co-producers in 6.8% [26]. Our cohort mirrored this distribution and exhibited a lower proportion (<1%) of non-carbapenemase CRE mechanisms than other regional reports (8.7%) [11], a finding consistent with Wise et al. [25]. Notably, our comparator group (non-CPE) consisted mainly of carbapenem-susceptible isolates, although it also included a small proportion of carbapenem-resistant strains without detectable carbapenemase genes. Thus, excess mortality cannot be attributed solely to carbapenemase production, representing an inherent limitation.

Furthermore, recent studies have shown that the zinc concentration in testing media can significantly influence the measured carbapenem MICs for NDM-producing *Enterobacterales* [27]. The Vitek 2 system used in our study employs media with supraphysiologic zinc levels (∼1.2 mg/L), which may artificially increase carbapenem MICs compared to physiological conditions [28]. Bilinskaya et al. and Asempa et al. demonstrated that lowering zinc concentrations restored meropenem MICs and improved the correlation with in vivo efficacy [28, 29]. Consequently, the relatively lower mortality observed among NDM-producing isolates in our cohort could partly reflect retained carbapenem activity despite the elevated in vitro MICs generated by zinc-rich testing systems.

The literature remains divided on whether carbapenemase production drives increased mortality. Hovan et al. reported higher mortality in CRE infections lacking carbapenemase genes (HR 2.4, 95% CI 1.2–4.6) [30], while Tamma et al. suggested increased virulence among CPE strains (aOR 3.19, 95% CI 0.99–10.25) [31], potentially mediated by siderophores, adhesins, and toxins [32]. In contrast, Baek et al., using propensity score adjustment, found no significant difference in mortality, suggesting that carbapenemase production may not be an independent predictor of outcome [33]. Notably, most prior studies were retrospective, had small sample sizes, and predominantly included KPC producers, limiting the generalizability to NDM-prevalent settings [34].

In our multivariable model, CPE infection remained independently associated with increased 30-day mortality (aHR 1.88, 95% CI 1.19–2.96). Among the CPE subtypes, KPC-producing *K. pneumoniae* conferred the highest risk (aHR 2.64), exceeding that of NDM-producing strains. These findings differ from those of Falcone et al., who reported a stronger association between mortality and metallo-β-lactamase (MBL) producers such as NDM (aOR 5.86, 95% CI 2.72–12.76), compared than in KPC (aOR 1.43, 95% CI 0.92–2.22) [5].

Supporting our results, Seo et al. reported higher mortality in patients infected with KPC- versus NDM-producing *Enterobacterales* in South Korea (34% vs 17%, *P* = .004) [35]. Their analysis identified bacteremia (HR 2.95, 95% CI 1.65–5.20) and pneumonia (HR 3.50, 95% CI 1.97–6.21) as key predictors of death. Similarly, KPC-producing *K. pneumoniae* had the highest risk (HR 2.19, 95% CI 1.21–3.95). Limited access to newer agents, such as ceftazidime/avibactam, meropenem/vaborbactam, or imipenem/relebactam [36, 37], as well as a higher burden of immunosuppression among KPC cases [38], may explain this trend.

Our study also included 4 cases of *E. coli* bacteremia due to NDM, with a 30-day mortality rate of 50%. There is limited evidence on clinical outcomes by carbapenemase subtype in *E. coli*. Boutzoukas et al. reported lower mortality among patients infected with MBL-producing *E. coli* (predominantly *bla*_NDM_) compared to those with non-MBL carbapenem-resistant strains (0% vs 26%, *P* = .020), although only three cases of bacteremia were reported [39]. In our cohort, no other carbapenemase types were detected in *E. coli*, precluding further species-specific comparison.

In the species-restricted analysis of *K. pneumoniae* BSI, the adjusted association between carbapenemase production and 30-day mortality was attenuated and not statistically significant. This finding aligns with recent studies showing that, after adjusting for illness severity and treatment adequacy, carbapenem resistance does not independently predict mortality. In a propensity score–matched cohort, Wang et al. reported a similar lack of association between carbapenem resistance and 30-day mortality (aHR 1.61; 95% CI 0.81–3.17) [40], while Giacobbe et al. observed that outcomes in patients with carbapenem-resistant *K. pneumoniae* were mainly influenced by host factors and delays in effective therapy rather than resistance mechanisms themselves [41].

A recent systematic review by Kanj et al. reported 30-day mortality associated with MBL infections ranging from 0% to 36.4% across six studies, mostly from Asia and Europe [36, 42]. However, differences in regional access to antibiotics such as aztreonam/avibactam and cefiderocol, as well as variations in clinical practices, limit the generalizability to Latin America. Our NDM subgroup had a higher mortality rate (45.2%), potentially reflecting the influence of COVID-19 pneumonia (25.8%), postsurgical comorbidity (46.8%), and higher initial severity of illness (median Pitt score: 5).

The study period overlapped with the first two years of the COVID-19 pandemic, during which in-hospital mortality in Peru reached unprecedented levels [43]. Approximately 20% of patients in the CPE group had COVID-19 comorbidity. However, after excluding these patients (*n* = 46), CPE infection remained independently associated with 30-day mortality (aHR 2.27, 95% CI 1.39–3.71), suggesting that the excess risk was not primarily driven by COVID-19 infection. Similar findings have been reported elsewhere: Pintado et al. observed high mortality among patients with CPE infections but no significant difference between COVID-19 and non-COVID-19 cases, indicating that outcomes may have been mainly determined by the intrinsic severity of KPC- and OXA-48–producing *K. pneumoniae* [44]. Other studies have also found that infection severity, rather than COVID-19 status, predicted the prognosis of patients with carbapenem-resistant infections [45, 46].

Studies focused on MBL-producing strains have consistently underscored clinical severity as a major determinant of outcomes. For instance, Anton-Vasquez et al. identified septic shock as a key mortality predictor (aOR 3.81, 95% CI 1.19–12.14) in a UK multicenter cohort with CPE bacteremia [47 ]. Herein, other predictors observed in our study, such as age ≥60 years (aOR 3.36, 95% CI 1.06–10.63) [48], and neutropenia (aHR 2.55, 95% CI 1.28–5.06), have also been associated with poor outcomes in previous research [49].

The higher mortality observed in patients with CPE BSI may reflect the interplay between early clinical severity and the intensity of supportive care rather than carbapenemase production itself [40, 41]. Variables such as the Pitt bacteremia score and ICU location are recognized mediators of this relationship, as they capture both the host's initial physiological derangement and the level of monitoring and organ support provided to the patient. Large propensity score studies on sepsis have shown that ICU management improves survival by enabling timely resuscitation and organ support, whereas delays or suboptimal monitoring in general wards increase mortality [50, 51]. Similarly, the EURECA multinational cohort demonstrated that adjustment for active therapy, source control, and illness severity (Pitt score) markedly attenuated the mortality hazard associated with CRE infections (HR decreasing from 2.57 to 1.41), supporting the role of early severity and care level as key outcome drivers [52]. Taken together, these observations highlight the importance of early recognition and appropriate supportive management of CPE infections. However, because our analysis was not intended to formally evaluate mediation, the extent to which early severity and level of care explain the mortality difference should be interpreted with caution.

Although concordant empirical therapy was associated with improved survival in bivariate analysis, the effect was not significant in the adjusted model. This is consistent with the findings of Satlin et al. [53], who reported persistent high 30-day mortality rate (49%), despite administration of active empirical therapy. This underscores the complexity of treating CPE infections and suggests that additional factors, such as bacterial burden [54], individual pharmacokinetics [55], or coexisting resistance mechanisms [56], may influence the outcomes.

Our study has several limitations. First, the retrospective design introduces potential selection and classification biases. Second, the reduced number of carbapenemase-producing *E. coli* isolates limits species-specific comparisons. Third, molecular characterization of carbapenemase variants was not performed, preventing the evaluation of genetic expression levels, presence of co-resistance mechanisms, or activity against newer agents. Additionally, therapeutic data reflect real-world clinical practice in resource-limited settings. Although empirical therapy is the strongest early determinant of outcome, subsequent targeted therapy may mitigate or amplify this effect, depending on the adequacy of antimicrobial adjustment. In our cohort, empirical therapy was analyzed separately to avoid collinearity, and the observed therapeutic patterns highlighted the practical limitations of optimizing treatment for CPE infections within our setting. Overall, these limitations prevent definitive genotype outcome associations but do not diminish the clinical relevance of our findings.

However, it is crucial to highlight that this is the first Peruvian study which assess the clinical impact of CPE BSI using molecular confirmation and confounder-adjusted analyses. While providing valuable local data, it offers new insight by examining the impact of carbapenemase subtypes, particularly in a setting with high NDM prevalence.

In conclusion, this study suggests that BSI caused by CPE are associated with significantly higher 30-day mortality compared to non-CPE infections, with KPC-producing *K. pneumoniae* posing the greatest risk. Our findings underscore the predominance of NDM, and the complex interplay between pathogen type, clinical severity, and patient outcomes. This study emphasizes the urgent need to strengthen molecular surveillance and ensure timely access to effective therapies, especially in NDM-endemic regions such as Peru.

## Supplementary Material

ofaf729_Supplementary_Data

## Supplementary Data

Supplementary materials are available at *Open Forum Infectious Diseases* online. Consisting of data provided by the authors to benefit the reader, the posted materials are not copyedited and are the sole responsibility of the authors, so questions or comments should be addressed to the corresponding author.

## References

[1] WHO publishes list of bacteria for which new antibiotics are urgently needed. Accessed 15 May 2025. Available at: https://www.who.int/news/item/27-02-2017-who-publishes-list-of-bacteria-for-which-new-antibiotics-are-urgently-needed

[2] Sati H , Carrara E, Savoldi A, et al The WHO bacterial priority pathogens list 2024: a prioritisation study to guide research, development, and public health strategies against antimicrobial resistance. Lancet Infect Dis 2025; doi: 10.1016/S1473-3099(25)00118-5. PMC12367593 40245910

[3] Antimicrobial Resistance Collaborators . Global burden of bacterial antimicrobial resistance in 2019: a systematic analysis. Lancet 2022; 399:629–55. 35065702 10.1016/S0140-6736(21)02724-0 PMC8841637

[4] Huang W , Qiao F, Zhang Y, et al In-hospital medical costs of infections caused by carbapenem-resistant *Klebsiella pneumoniae*. Clin Infect Dis 2018; 67:S225–30. 30423052 10.1093/cid/ciy642

[5] Falcone M , Tiseo G, Carbonara S, et al Mortality attributable to bloodstream infections caused by different carbapenem-resistant gram-negative bacilli: results from a nationwide study in Italy (ALARICO network). Clin Infect Dis 2023; 76:2059–69. 36801828 10.1093/cid/ciad100

[6] Meletis G , Chatzidimitriou D, Malisiovas N. Double- and multi-carbapenemase-producers: the excessively armored bacilli of the current decade. Eur J Clin Microbiol Infect Dis 2015; 34:1487–93. 25894987 10.1007/s10096-015-2379-9

[7] Allel K , Peters A, Conejeros J, et al Antibiotic consumption during the coronavirus disease 2019 pandemic and emergence of carbapenemase-producing *Klebsiella pneumoniae* lineages among inpatients in a Chilean hospital: a time-series study and phylogenomic analysis. Clin Infect Dis 2023; 77:S20–8. 37406053 10.1093/cid/ciad151 PMC10321701

[8] Kiffer CRV , Rezende TFT, Costa-Nobre DT, et al A 7-year Brazilian national perspective on plasmid-mediated carbapenem resistance in *Enterobacterales*, *Pseudomonas aeruginosa*, and *Acinetobacter baumannii* complex and the impact of the coronavirus disease 2019 pandemic on their occurrence. Clin Infect Dis 2023; 77:S29–37. 37406041 10.1093/cid/ciad260 PMC10321697

[9] Thomas GR , Corso A, Pasterán F, et al Increased detection of carbapenemase-producing *Enterobacterales* bacteria in Latin America and the Caribbean during the COVID-19 pandemic. Emerg Infect Dis 2022; 28:1–8. 10.3201/eid2811.220415 PMC9622262 36286547

[10] Boyd SE , Livermore DM, Hooper DC, Hope WW. Metallo-β-lactamases: structure, function, epidemiology, treatment options, and the development pipeline. Antimicrob Agents Chemother 2020; 64:e00397–20. 32690645 10.1128/AAC.00397-20 PMC7508574

[11] Villegas MV , Pallares CJ, Escandón-Vargas K, et al Characterization and clinical impact of bloodstream infection caused by carbapenemase-producing Enterobacteriaceae in seven Latin American countries. PLoS One 2016; 11:e0154092. 27104910 10.1371/journal.pone.0154092 PMC4841576

[12] Krapp F , Cuicapuza D, Salvatierra G, et al Emerging carbapenem-resistant *Klebsiella pneumoniae* in a tertiary care hospital in Lima, Peru. Microbiol Spectr 2025; 13:e0182524. 39792003 10.1128/spectrum.01825-24 PMC11792469

[13] Angles-Yanqui E , Huaringa-Marcelo J, Sacsaquispe-Contreras R, Pampa-Espinoza L. Panorama de las carbapenemasas en Perú. Rev Panam Salud Publica 2020; 44:e61. 32973907 10.26633/RPSP.2020.61 PMC7498286

[14] Krapp F , García C, Hinostroza N, et al Prevalence of antimicrobial resistance in gram-negative bacteria bloodstream infections in Peru and associated outcomes: VIRAPERU study. Am J Trop Med Hyg 2023; 109:1095–106. 37722663 10.4269/ajtmh.22-0556 PMC10622474

[15] CDC/NHSN Surveillance Definitions for Specific Types of Infections. Accessed 15 May 2025. Available at: https://www.cdc.gov/nhsn/pdfs/pscmanual/17pscnosinfdef_current.pdf

[16] OpenEpi: Sample Size for X-Sectional, Cohort, and Clinical Trials. Accessed 1 May 2025. Available at: https://www.openepi.com/SampleSize/SSCohort.htm

[17] Gutiérrez-Gutiérrez B , Salamanca E, de Cueto M, et al Effect of appropriate combination therapy on mortality of patients with bloodstream infections due to carbapenemase-producing Enterobacteriaceae (INCREMENT): a retrospective cohort study. Lancet Infect Dis 2017; 17:726–34. 28442293 10.1016/S1473-3099(17)30228-1

[18] Hoellinger B , Deboscker S, Danion F, et al Incidence and time-to-onset of carbapenemase-producing *Enterobacterales* (CPE) infections in CPE carriers: a retrospective cohort study. Microbiol Spectr 2022; 10:e0186822. 36321906 10.1128/spectrum.01868-22 PMC9769894

[19] Kitaya S , Kanamori H, Baba H, et al Clinical and epidemiological characteristics of persistent bacteremia: a decadal observational study. Pathogens 2023; 12:212. 36839484 10.3390/pathogens12020212 PMC9960527

[20] Spivak ES , Cosgrove SE, Srinivasan A. Measuring appropriate antimicrobial use: attempts at opening the black box. Clin Infect Dis 2016; 63:1639–44. 27682070 10.1093/cid/ciw658 PMC6487652

[21] Seo H , Lee SC, Chung H, et al Clinical and microbiological analysis of risk factors for mortality in patients with carbapenem-resistant Enterobacteriaceae bacteremia. Int J Antimicrob Agents 2020; 56:106126. 32755654 10.1016/j.ijantimicag.2020.106126

[22] Lai C , Ma Z, Zhang J, et al Efficiency of combination therapy versus monotherapy for the treatment of infections due to carbapenem-resistant gram-negative bacteria: a systematic review and meta-analysis. Syst Rev 2024; 13:309. 39702227 10.1186/s13643-024-02695-x PMC11658076

[23] Clinical and Laboratory Standards Institute (CLSI) . Performance standards for antimicrobial susceptibility testing. 34th ed. CLSI supplement M100 (ISBN 978-1-68440-220-5 [print]; ISBN 978-1-68440-221-2 [electronic]). USA: Clinical and Laboratory Standards Institute, 2024.

[24] Budhram DR , Mac S, Bielecki JM, Patel SN, Sander B. Health outcomes attributable to carbapenemase-producing Enterobacteriaceae infections: a systematic review and meta-analysis. Infect Control Hosp Epidemiol 2020; 41:37–43. 31637986 10.1017/ice.2019.282

[25] Wise MG , Karlowsky JA, Mohamed N, et al Global trends in carbapenem- and difficult-to-treat-resistance among World Health Organization priority bacterial pathogens: ATLAS surveillance program 2018–2022. J Glob Antimicrob Resist 2024; 37:168–75. 38608936 10.1016/j.jgar.2024.03.020

[26] Reyes Quevedo YV , Astocondor AL, Riveros MD, et al P-1372. Distribution and mechanisms of resistance among carbapenem-resistant *Enterobacterales* in Peru. Open Forum Infect Dis 2025; 12:ofae631–1549.

[27] Abdelraouf K , Gill CM, Gethers M, et al Deciphering the efficacy of β-lactams in the face of metallo-β-lactamase-derived resistance in *Enterobacterales*: supraphysiologic zinc in the broth is the culprit. Open Forum Infect Dis 2024; 11:ofae228. 38813259 10.1093/ofid/ofae228 PMC11134298

[28] Asempa TE , Gill CM, Chibabhai V, Nicolau DP. Comparison of zinc concentrations in the broth of commercial automated susceptibility testing devices (Vitek 2, MicroScan, BD Phoenix, and Sensititre). Microbiol Spectr 2022; 10:e0005222. 35377221 10.1128/spectrum.00052-22 PMC9045177

[29] Bilinskaya A , Buckheit DJ, Gnoinski M, Asempa TE, Nicolau DP. Variability in zinc concentration among Mueller-Hinton broth brands: impact on antimicrobial susceptibility testing of metallo-β-lactamase-producing Enterobacteriaceae. J Clin Microbiol 2020; 58:e02019–20. 32999009 10.1128/JCM.02019-20 PMC7685897

[30] Hovan MR , Narayanan N, Cedarbaum V, Bhowmick T, Kirn TJ. Comparing mortality in patients with carbapenemase-producing carbapenem resistant *Enterobacterales* and non-carbapenemase-producing carbapenem resistant *Enterobacterales* bacteremia. Diagn Microbiol Infect Dis 2021; 101:115505. 34399381 10.1016/j.diagmicrobio.2021.115505

[31] Tamma PD , Goodman KE, Harris AD, et al Comparing the outcomes of patients with carbapenemase-producing and non-carbapenemase-producing carbapenem-resistant Enterobacteriaceae bacteremia. Clin Infect Dis 2017; 64:257–64. 28013264 10.1093/cid/ciw741 PMC5241781

[32] Jomehzadeh N , Jahangirimehr F, Chegeni SA. Virulence-associated genes analysis of carbapenemase-producing *Escherichia coli* isolates. PLoS One 2022; 17:e0266787. 35536848 10.1371/journal.pone.0266787 PMC9089865

[33] Baek MS , Kim JH, Park JH, Kim TW, Jung HI, Kwon YS. Comparison of mortality rates in patients with carbapenem-resistant *Enterobacterales* bacteremia according to carbapenemase production: a multicenter propensity-score matched study. Sci Rep 2024; 14:597. 38182719 10.1038/s41598-023-51118-9 PMC10770160

[34] Suay-García B , Pérez-Gracia MT. Present and future of carbapenem-resistant Enterobacteriaceae (CRE) infections. Antibiotics (Basel) 2019; 8:122. 31430964 10.3390/antibiotics8030122 PMC6784177

[35] Seo H , Kim HJ, Kim MJ, et al Comparison of clinical outcomes of patients infected with KPC- and NDM-producing *Enterobacterales*: a retrospective cohort study. Clin Microbiol Infect 2021; 27:1167.e1–.e8. 10.1016/j.cmi.2020.09.043 33010443

[36] Tamma PD , Heil EL, Justo JA, Mathers AJ, Satlin MJ, Bonomo RA. Infectious Diseases Society of America 2024 guidance on the treatment of antimicrobial-resistant gram-negative infections. Clin Infect Dis 2024; doi:10.1093/cid/ciae403 39108079

[37] Balbuena JP , Cordova E, Mykietiuk A, et al Carbapenem-resistant gram-negative bacilli bacteremia in Argentina (EMBARCAR): findings from a prospective, multicenter cohort study. Clin Infect Dis 2025; doi: 10.1093/cid/ciaf259. 40393137

[38] Santoro A , Franceschini E, Meschiari M, et al Epidemiology and risk factors associated with mortality in consecutive patients with bacterial bloodstream infection: impact of MDR and XDR Bacteria. Open Forum Infect Dis 2020; 7:ofaa461. 33209951 10.1093/ofid/ofaa461 PMC7652098

[39] Boutzoukas AE , Komarow L, Chen L, et al International epidemiology of carbapenemase-producing *Escherichia coli*. Clin Infect Dis 2023; 77:499–509. 37154071 10.1093/cid/ciad288 PMC10444003

[40] Wang L , Zeng C, Li X, Li Y, Liu Z, Hu J. Mortality associated with carbapenem resistance in *Klebsiella pneumoniae* bloodstream infection: a propensity score-matched study. Infect Control Hosp Epidemiol 2024; 45:839–46. 38487826 10.1017/ice.2024.21

[41] Giacobbe DR , Marelli C, Cattardico G, et al Mortality in KPC-producing *Klebsiella pneumoniae* bloodstream infections: a changing landscape. J Antimicrob Chemother 2023; 78:2505–14. 37606528 10.1093/jac/dkad262

[42] Kanj SS , Kantecki M, Arhin FF, Gheorghe M. Epidemiology and outcomes associated with MBL-producing *Enterobacterales*: a systematic literature review. Int J Antimicrob Agents 2025; 65:107449. 39884321 10.1016/j.ijantimicag.2025.107449

[43] Mas-Ubillus G , Ortiz PJ, Huaringa-Marcelo J, et al High mortality among hospitalized adult patients with COVID-19 pneumonia in Peru: a single centre retrospective cohort study. PLoS One 2022; 17:e0265089. 35259196 10.1371/journal.pone.0265089 PMC8903290

[44] Pintado V , Ruiz-Garbajosa P, Escudero-Sanchez R, et al Carbapenemase-producing *Enterobacterales* infections in COVID-19 patients. Infect Dis (Lond) 2022; 54:36–45. 34382910 10.1080/23744235.2021.1963471 PMC8425444

[45] Falasca K , Vetrugno L, Borrelli P, et al Antimicrobial resistance in intensive care patients hospitalized with SEPSIS: a comparison between the COVID-19 pandemic and pre-pandemic era. Front Med (Lausanne) 2024; 11:1355144. 38813381 10.3389/fmed.2024.1355144 PMC11133528

[46] Al Bshabshe A , Hamid ME, Salem E, et al The extent of carbapenem-resistant encoding genes in *Klebsiella pneumoniae* from COVID-19 and non-COVID-19 patients in a tertiary care center, Saudi Arabia. Braz J Med Biol Res 2025; 58:e14066. 40136226 10.1590/1414-431X2025e14066

[47] Anton-Vazquez V , Evans TJ, Fernando S, et al Clinical, microbiological characteristics and predictors of mortality in patients with carbapenemase-producing *Enterobacterales* bloodstream infections: a multicentre study. Infect Prev Pract 2023; 5:100298. 37534297 10.1016/j.infpip.2023.100298 PMC10393540

[48] Zhao S , Kennedy S, Perry MR, et al Epidemiology of and risk factors for mortality due to carbapenemase-producing organisms (CPO) in healthcare facilities. J Hosp Infect 2021; 110:184–93. 33571557 10.1016/j.jhin.2021.01.028 PMC8035079

[49] Karnmueng P , Montakantikul P, Paiboonvong T, Plongla R, Chatsuwan T, Chumnumwat S. Mortality factors and antibiotic options in carbapenem-resistant *Enterobacterales* bloodstream infections: insights from a high-prevalence setting with co-occurring NDM-1 and OXA-48. Clin Transl Sci 2024; 17:e13855. 38853376 10.1111/cts.13855 PMC11163016

[50] Oami T , Imaeda T, Nakada TA, et al Mortality analysis among sepsis patients in and out of intensive care units using the Japanese nationwide medical claims database: a study by the Japan Sepsis Alliance study group. J Intensive Care 2023; 11:2. 36611188 10.1186/s40560-023-00650-x PMC9826578

[51] Ahn YH , Lee J, Oh DK, et al Association between the timing of ICU admission and mortality in patients with hospital-onset sepsis: a nationwide prospective cohort study. J Intensive Care 2023; 11:16. 37085923 10.1186/s40560-023-00663-6 PMC10120484

[52] Paniagua-García M , Bravo-Ferrer JM, Pérez-Galera S, et al Attributable mortality of infections caused by carbapenem-resistant *Enterobacterales*: results from a prospective, multinational case-control-control matched cohorts study (EURECA). Clin Microbiol Infect 2024; 30:223–30. 38267096 10.1016/j.cmi.2023.11.008

[53] Satlin MJ , Chen L, Patel G, et al Multicenter clinical and molecular epidemiological analysis of bacteremia due to carbapenem-resistant Enterobacteriaceae (CRE) in the CRE epicenter of the United States. Antimicrob Agents Chemother 2017; 61:e02349-16. 28167547 10.1128/AAC.02349-16 PMC5365653

[54] Li J , Xie S, Ahmed S, et al Antimicrobial activity and resistance: influencing factors. Front Pharmacol 2017; 8:364. 28659799 10.3389/fphar.2017.00364 PMC5468421

[55] Adembri C , Cappellini I, Novelli A. The role of PK/PD–based strategies to preserve new molecules against multi-drug resistant gram-negative strains. J Chemother 2020; 32:219–25. 32628094 10.1080/1120009X.2020.1786634

[56] Mmatli M , Mbelle NM, Maningi NE, Osei Sekyere J. Emerging transcriptional and genomic mechanisms mediating carbapenem and polymyxin resistance in Enterobacteriaceae: a systematic review of current reports. mSystems 2020; 5:10.1128/msystems.00783-20. 10.1128/mSystems.00783-20 PMC7771540 33323413

