# Supplementary material for: Thirty-Day Mortality Associated With Carbapenemase-Producing Enterobacterales Bloodstream Infections at a Referral Hospital in Peru, 2020–2023
Source: Open Forum Infect Dis. 2025 Dec 1;12(12):ofaf729. doi: 10.1093/ofid/ofaf729 (PMC12692351; doi:10.1093/ofid/ofaf729)
Supplement: ofaf729_Supplementary_Data [file ofaf729_supplementary_data.zip › Supplemental_material.docx]

**Supplementary data**

**Figure S1.** Directed acyclic graph (DAG) illustrating hypothesized causal relationships between carbapenemase-producing *Enterobacterales* (CPE) bloodstream infection diagnosis and 30-day all-cause mortality. The node “CPE BSI diagnosis” represents exposure. The mediators included ICU admission at onset, Pitt bacteremia score, and targeted antimicrobial therapy. Confounders included age, immunosuppression, pneumonia as an infection source, septic shock, and concordant empirical antimicrobial therapy. This model was used to inform the covariate selection for the multivariable analysis.


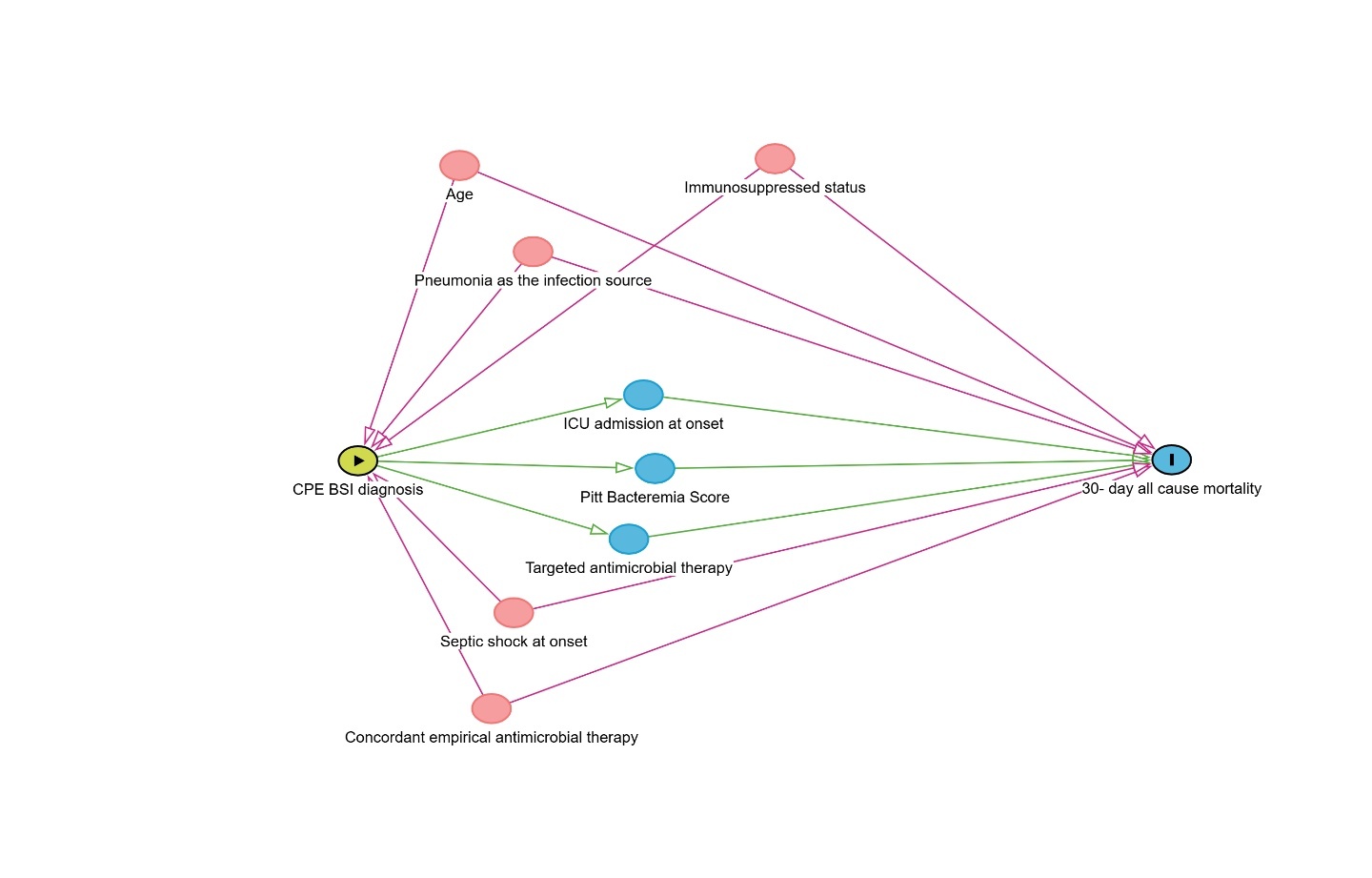


**Figure S2.** Forest plot of adjusted hazard ratios (aHRs) for 30-day all-cause mortality associated with carbapenemase-producing *Enterobacterales* (CPE) bloodstream infection compared with non-CPE. The dashed vertical line at HR = 1.0 represents the reference (non-CPE) group. Blue points correspond to the main cohort, red to the cohort excluding COVID-19 patients, and green to the *Klebsiella pneumoniae*-only subgroup. Horizontal bars indicate 95% confidence intervals on a logarithmic scale. Across analyses, CPE infection was consistently associated with higher mortality, although the effect did not reach statistical significance in the K. pneumoniae subgroup, likely reflecting the smaller sample size and greater baseline severity.


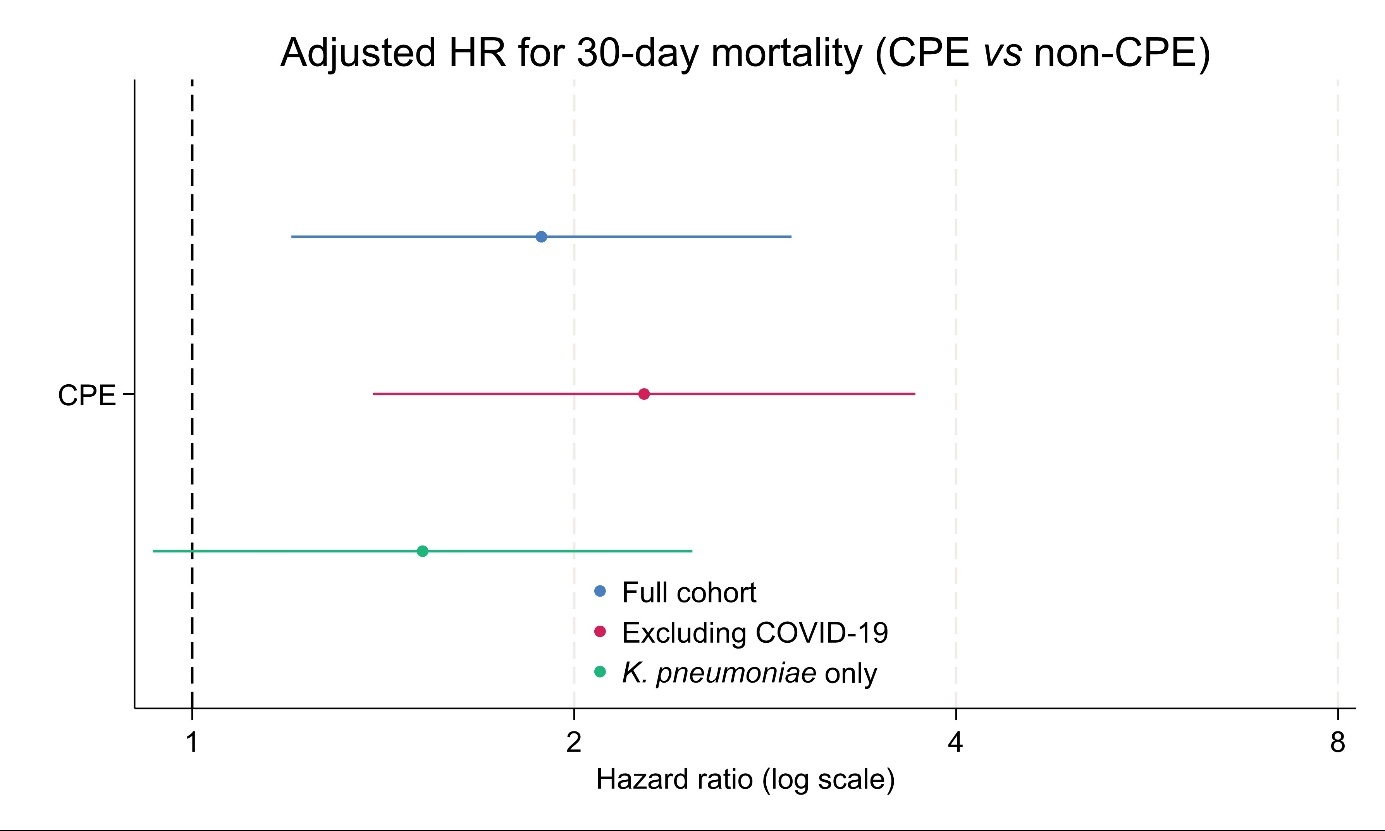


**Table S1.** Assessment of Proportional Hazards Assumption (Schoenfeld Residuals)

| Variable | rho | Chi² | P value |
| --- | --- | --- | --- |
| Pathogen type (CPE vs non-CPE) | -0.00775 | 0.01 | 0.9215 |
| Age ≥60 years | -0.06785 | 0.61 | 0.4347 |
| Source: Pneumonia | 0.11347 | 2.10 | 0.1475 |
| Septic shock at onset | -0.14748 | 3.23 | 0.0721 |
| Concordant empirical therapy | -0.02755 | 0.11 | 0.7380 |
| Immunosuppressed | -0.05626 | 0.43 | 0.5136 |
| Global test |  | 6.32 | 0.3878 |

CPE = carbapenemase-producing *Enterobacterales*

## Table S2. Distribution of Bloodstream Infection Episodes by Comorbidities and Risk Factors

| Variable | CPE  (n = 97) | Non-CPE  (n = 409) | *P* value |
| --- | --- | --- | --- |
| Comorbidities, n (%) |  |  |  |
| Diabetes mellitus | 20 (20.6) | 100 (24.5) | 0.425 |
| Chronic kidney disease | 17 (17.5) | 106 (25.9) | 0.083 |
| Post-surgical status | 35 (36.1) | 90 (22.0) | 0.004 |
| COVID-19 | 19 (19.6) | 27 (6.6) | <0.001 |
| Chronic obstructive pulmonary disease | 1 (1.0) | 12 (2.9) | 0.478 |
| Liver disease | 5 (5.2) | 49 (11.9) | 0.050 |
| HIV/AIDS | 3 (3.1) | 11 (2.7) | 0.738 |
| Cerebrovascular disease | 8 (8.3) | 31 (7.6) | 0.825 |
| Acute myocardial infarction | 2 (2.1) | 7 (1.7) | 0.685 |
| Peripheral vascular disease | 7 (7.2) | 24 (5.9) | 0.638 |
| Chronic heart failure | 5 (5.2) | 27 (6.6) | 0.599 |
| Hypertension | 27 (27.8) | 143 (34.9) | 0.181 |
| Connective tissue disease | 4 (4.1) | 7 (1.7) | 0.235 |
| Burns | 2 (2.1) | 0 (0.0) | 0.036 |
| Tuberculosis | 1 (1.0) | 11 (2.7) | 0.477 |
| Obesity | 15 (15.5) | 23 (5.6) | 0.001 |
| Prior IV antibiotic use (last 30 days) | 40 (41.2) | 142 (34.7) | 0.229 |
| Hospitalization in past 90 days | 57 (58.8) | 217 (53.1) | 0.311 |
| ICU admission in past 90 days | 11 (11.3) | 14 (3.4) | 0.003 |
| Mechanical ventilation | 58 (59.8) | 61 (14.9) | <0.001 |
| Central venous catheter | 89 (91.8) | 205 (50.1) | <0.001 |
| Urinary catheter | 65 (67.0) | 150 (36.7) | <0.001 |
| Vasopressor use | 41 (42.3) | 93 (22.7) | <0.001 |
| Immunosuppressive therapy | 20 (20.6) | 61 (14.9) | 0.168 |
| Hemodialysis | 13 (13.4) | 67 (16.4) | 0.470 |
| Source of bacteremia, n (%) |  |  |  |
| Urinary tract | 14 (14.4) | 145 (35.5) | <0.001 |
| Catheter-related | 15 (15.5) | 47 (11.5) | 0.283 |
| Pneumonia | 38 (39.2) | 52 (12.7) | <0.001 |
| Intra-abdominal | 15 (15.5) | 114 (27.9) | 0.012 |
| Skin and soft tissue | 7 (7.2) | 19 (4.7) | 0.302 |
| Meningeal | 3 (3.1) | 4 (1.0) | 0.133 |
| Unknown | 3 (3.1) | 22 (5.4) | 0.443 |
| Endocarditis | 0 (0) | 2 (0.5) | 1.000 |
| Osteoarticular | 0 (0) | 1 (0.2) | 1.000 |
| Other | 2 (2.1) | 3 (0.7) | 0.245 |

Abbreviations: CPE, carbapenemase-producing *Enterobacterales*; ICU, intensive care unit; COPD, chronic obstructive pulmonary disease; HIV, human immunodeficiency virus; AIDS, acquired immunodeficiency syndrome; IV, intravenous.

**Table S3.** Distribution of Bloodstream Infection Episodes by Subgroup

| Variable | KPC  (n = 35) | NDM  (n = 62) | Non-CPE  (n = 409) | *P* value |
| --- | --- | --- | --- | --- |
| Sex |  |  |  |  |
| Male, n (%) | 21 (60.0) | 44 (70.9) | 229 (55.9) | 0.081 |
| Age, median (IQR) | 57 (45–66) | 56 (42–68) | 66 (52–76) | <0.001 |
| Hospital ward, n (%) |  |  |  |  |
| ICU | 9 (25.7) | 44 (70.9) | 49 (12.0) | <0.001 |
| Medical ward | 15 (42.9) | 8 (12.9) | 122 (29.8) | 0.004 |
| Surgical ward | 7 (20.0) | 4 (6.5) | 37 (9.1) | 0.072 |
| Emergency department | 4 (11.4) | 6 (9.7) | 201 (49.1) | <0.001 |
| Comorbidities, n (%) |  |  |  |  |
| Diabetes mellitus | 5 (14.3) | 15 (24.2) | 100 (24.5) | 0.397 |
| Chronic kidney disease | 7 (20.0) | 10 (16.1) | 106 (25.9) | 0.204 |
| Post-surgical status | 6 (17.1) | 29 (46.8) | 90 (22.0) | <0.001 |
| COVID-19 | 3 (8.6) | 16 (25.8) | 27 (6.6) | <0.001 |
| Immunosuppression, n (%) |  |  |  |  |
| Chemotherapy (last 6 months) | 7 (20.0) | 2 (3.2) | 58 (14.2) | 0.028 |
| Solid organ transplant | 5 (14.3) | 2 (3.2) | 13 (3.2) | 0.018 |
| Bone marrow transplant (past year) | 1 (2.9) | 0 (0.0) | 2 (0.5) | 0.232 |
| Chronic corticosteroid therapy | 3 (8.6) | 3 (4.8) | 26 (6.4) | 0.732 |
| Neutropenia <200 cells/μL | 6 (17.1) | 1 (1.6) | 42 (10.3) | 0.030 |
| Charlson comorbidity index, median (IQR) | 4 (2–6) | 3.5 (1–5) | 5 (3–6) | 0.002 |
| Source of bacteremia, n (%) |  |  |  |  |
| Urinary tract | 6 (17.1) | 8 (12.9) | 145 (35.5) | <0.001 |
| Catheter-related | 3 (8.6) | 12 (19.4) | 47 (11.5) | 0.168 |
| Pneumonia | 10 (28.6) | 28 (45.2) | 52 (12.7) | <0.001 |
| Intra-abdominal | 8 (22.8) | 7 (11.3) | 114 (27.9) | 0.019 |
| Skin and soft tissue | 4 (11.4) | 3 (4.8) | 19 (4.7) | 0.217 |
| Pitt bacteremia score, median (IQR) | 4 (2–6) | 5 (3–6) | 2 (0–3) | <0.001 |
| Septic shock at onset, n (%) | 16 (45.7) | 29 (46.8) | 109 (26.7) | <0.001 |
| ICU admission on day 1, n (%) | 13 (37.1) | 49 (79.0) | 57 (13.9) | <0.001 |
| Pathogen, n (%) |  |  |  | <0.001 |
| *Klebsiella pneumoniae* | 35 (100.0) | 58 (93.5) | 140 (34.2) |  |
| *Escherichia coli* | 0 (0.0) | 4 (6.5) | 269 (65.8) |  |

Abbreviations: IQR, interquartile range; ICU, intensive care unit; CPE, carbapenemase-producing *Enterobacterales*.

## Table S4. Multivariable Analysis of 30-Day Mortality in Patients with *Enterobacterales* Bacteremia by Pathogen Subtype

| Variable | aHR | 95% CI | P value |
| --- | --- | --- | --- |
| Age ≥60 years (vs <60) | 1.48 | 1.03–2.13 | 0.034 |
| Septic shock at onset | 2.93 | 2.06–4.17 | <0.001 |
| Source of bacteremia |  |  |  |
| No pneumonia (Ref.) |  |  |  |
| Pneumonia | 1.78 | 1.20–2.65 | 0.004 |
| Concordant empirical antibiotic therapy | 0.72 | 0.48–1.09 | 0.130 |
| Immunocompromised status | 1.59 | 1.09–2.31 | 0.014 |
| Pathogen type |  |  |  |
| Non-CPE (Ref.) |  |  |  |
| KPC | 2.64 | 1.52–4.59 | 0.001 |
| NDM | 1.50 | 0.89–2.54 | 0.129 |

Abbreviations: aHR, adjusted hazard ratio; CI, confidence interval; CPE, carbapenemase-producing *Enterobacterales*; NDM, New Delhi metallo-β-lactamase. Multivariable analysis adjusted for all variables listed in the table.

## Subgroup Analysis: Mortality Comparison Between KPC and NDM Pathogens

| Variable | aHR | 95% CI | P value |
| --- | --- | --- | --- |
| NDM (vs KPC, Ref.) | 0.57 | 0.32–1.01 | 0.055 |

## Abbreviations: aHR, adjusted hazard ratio; CI, confidence interval; CPE, carbapenemase-producing *Enterobacterales*; NDM, New Delhi metallo-β-lactamase. Multivariable analysis adjusted for all variables listed in the table.

**Table S5.** Sensitivity Analysis: Multivariable Cox Regression for 30-Day All-Cause Mortality Excluding Patients with COVID-19

| Variable | aHR | 95% CI | P value |
| --- | --- | --- | --- |
| Age ≥60 years (vs <60) | 1.59 | 1.07–2.36 | 0.022 |
| Septic shock at onset | 2.97 | 2.07–4.27 | <0.001 |
| Source of bacteremia |  |  |  |
| No pneumonia (Ref.) |  |  |  |
| Pneumonia | 2.06 | 1.35–3.15 | 0.001 |
| Concordant empirical antibiotic therapy | 0.82 | 0.52–1.28 | 0.378 |
| Immunocompromised status | 1.55 | 1.07–2.26 | 0.022 |
| Pathogen type |  |  |  |
| Non-CPE (Ref.) |  |  |  |
| CPE | 2.27 | 1.39–3.71 | 0.001 |

Abbreviations: aHR, adjusted hazard ratio; CI, confidence interval; CPE, carbapenemase-producing *Enterobacterales*. Multivariate analysis adjusted for all variables listed in the table.

**Table S6.** Baseline characteristics and adjusted analysis of factors associated with 30-day mortality in patients with *K. pneumoniae* bloodstream infection

| Baseline characteristics | CPE  (n=93) | Non-CPE  (n=140) | *P* value |
| --- | --- | --- | --- |
| Age ≥60 years, n (%) | 42 (45.2) | 86 (61.4) | 0.015 |
| Immunosuppression, n (%) | 18 (19.4) | 26 (18.6) | 0.881 |
| Pneumonia source, n (%) | 37 (39.8) | 34 (24.3) | 0.012 |
| Septic shock at onset, n (%) | 43 (46.2) | 41 (29.3) | 0.008 |
| ICU admission on day 1, n (%) | 61 (65.6) | 29 (20.9) | <0.001 |
| Appropriate empiric therapy, n (%) | 21 (22.6) | 106 (75.7) | <0.001 |
| 30-day mortality, n (%) | 49 (52.7) | 39 (27.9) | <0.001 |
|  |  |  |  |
|  |  |  |  |
| Multivariable Cox model | **aHR (95% CI)** | ***P* value** |  |
| CPE infection | 1.44 (0.84–2.47) | 0.189 |  |
| Age ≥60 years | 1.34 (0.87–2.06) | 0.184 |  |
| Pneumonia source | 1.57 (0.99–2.50) | 0.057 |  |
| Septic shock at onset | 2.45 (1.57–3.83) | <0.001 |  |
| Appropriate empiric therapy | 0.62 (0.37–1.06) | 0.079 |  |
| Immunosuppression | 1.98 (1.25–3.13) | 0.004 |  |

Abbreviations: CPE, carbapenemase-producing *Enterobacterales*; HR, hazard ratio; CI, confidence interval; IQR, interquartile range; ICU, intensive care unit.

Variables included in the model are listed above.

## Table S7. Outcomes of Carbapenemase-Producing *Enterobacterales* (CPE) Bacteremia According to Definitive Antimicrobial Regimen

| **Definitive antimicrobial regimen** | **Patients, n** | **30-day mortality,**  **n (%)** | **NDM (n)** | **KPC (n)** |
| --- | --- | --- | --- | --- |
| **Colistin-based regimens** | 68 | 30 (44.1) | 47 | 21 |
| Colistin + Tigecycline | 39 | 17 (43.6) | 30 | 9 |
| Colistin + Carbapenem | 20 | 10 (50.0) | 12 | 8 |
| Colistin + Tigecycline + Carbapenem | 3 | 1 (33.3) | 3 | – |
| Colistin + Tigecycline + Aminoglycoside | 1 | 0 (0.0) | 1 | – |
| Colistin + Carbapenem + Aminoglycoside | 2 | 0 (0.0) | 1 | 1 |
| Colistin + Tigecycline + Quinolone | 1 | 1 (100.0) | – | 1 |
| Colistin + Quinolone | 2 | 1 (50.0) | – | 2 |
| **Other combinations** | 3 | 1 (33.3) | 2 | 1 |
| Ceftazidime/avibactam + Aztreonam | 1 | 1 (100.0) | 1 | – |
| Carbapenem + Aminoglycoside | 1 | 0 (0.0) | 1 | – |
| Carbapenem + Tigecycline + Aminoglycoside | 1 | 0 (0.0) | – | 1 |
| **Monotherapy** | 8 | 2 (25.0) | 5 | 3 |
| Ceftazidime/Avibactam | 1 | 0 (0.0) | – | 1 |
| Aminoglycoside | 3 | 0 (0.0) | 2 | 1 |
| Colistin | 4 | 2 (50.0) | 3 | 1 |

Abbreviations: CPE, carbapenemase-producing *Enterobacterales*; NDM, New Delhi metallo-β-lactamase; KPC, *Klebsiella pneumoniae* carbapenemase.

Mortality data correspond to 30-day all-cause mortality. This table includes only patients from the CPE group who received definitive therapy.

**Table S8.** Antimicrobial Susceptibility in Patients with CPE and Non-CPE Bacteremia

| **Antibiotic** | **CPE (S)** | **CPE (I)** | **CPE (R)** | **Non-CPE (S)** | **Non-CPE (I)** | **Non-CPE (R)** |
| --- | --- | --- | --- | --- | --- | --- |
| Ertapenem | 0  (0.0%) | 0  (0.0%) | 97  (100%) | 405  (99.1%) | 3  (0.7%) | 1  (0.2%) |
| Meropenem | 1  (1.0%) | 1  (1.0%) | 95  (98.0%) | 408  (99.8%) | 1  (0.2%) | 0  (0.0%) |
| Imipenem | 0  (0.0%) | 0  (0.0%) | 97  (100.0%) | 407  (99.6%) | 1  (0.2%) | 1  (0.2%) |
| Cefazolin | 0/96  (0.0%) | 0/96  (0.0%) | 96/96  (100%) | 125/383 (32.6%) | 6/383  (1.6%) | 252/383  (65.8%) |
| Cefepime¹ | 2  (2.1%) | 0  (0.0%) | 95  (97.9%) | 167  (40.8%) | 0  (0.0%) | 242  (59.2%) |
| Ceftriaxone | 0  (0.0%) | 0  (0.0%) | 97  (100%) | 153/406 (37.7%) | 6/406  (1.5%) | 247/406  (60.8%) |
| Ceftazidime | 0  (0.0%) | 1  (1.0%) | 96  (98.9%) | 159  (38.9%) | 7  (1.7%) | 243  (59.4%) |
| Aztreonam | 0  (0.0%) | 0  (0.0%) | 97  (100%) | 157/406 (38.7%) | 3/406  (0.7%) | 246/406  (60.6%) |
| Ceftazidime/avibactam | 39  (40.2%) | ─ | 58  (59.8%) | 263/264 (99.6%) | ─ | 1/264  (0.4%) |
| Piperacillin/tazobactam¹ | 0  (0.0%) | 0  (0.0%) | 97  (100%) | 335/408 (82.1%) | 31/408  (7.6%) | 42/408  (10.3%) |
| Ampicillin/sulbactam | 0  (0.0%) | 0  (0.0%) | 97  (100%) | 144/409 (35.2%) | 37/409  (9.1%) | 228/409  (55.8%) |
| Ceftolozane/tazobactam | 2  (2.1%) | 0  (0.0%) | 95  (97.9%) | 244/265 (92.1%) | 5/265  (1.9%) | 16/265  (6.0%) |
| Amikacin | 90  (92.8%) | 0  (0.0%) | 7  (7.2%) | 389/409 (95.1%) | 2/409  (0.5%) | 18/409  (4.4%) |
| Gentamicin | 6  (6.2%) | 0  (0.0%) | 91  (93.8%) | 149/381 (39.1%) | 3/381  (0.8%) | 229/381  (60.1%) |
| Ciprofloxacin | 4  (4.1%) | 0  (0.0%) | 93  (95.9%) | 94/409  (23.0%) | 40/409  (9.8%) | 275/409  (67.2%) |
| Trimethoprim/sulfamethoxazole | 3  (3.1%) | ─ | 94  (96.9%) | 100/383 (26.1%) | ─ | 283/383  (73.9%) |
| Colistin^2^ | ─ | 91  (93.8%) | 6  (6.2%) | ─ | 271/274  (98.9%) | 3/274  (1.1%) |
| Tigecycline* | 55  (56.7%) | ─ | 42  (43.3%) | 264/286 (92.3%) | ─ | 22/286  (7.7%) |

Abbreviations: Data are presented as n (%) representing susceptibility patterns of *Enterobacterales* isolates in each group.

¹ SDD: Susceptible-Dose Dependent, considered in place of intermediate category.

^2^ Colistin was categorized as intermediate, as no susceptible category exists.

* EUCAST 2019 breakpoints for *E. coli* and *K. pneumoniae*.

S = susceptible; I = intermediate; R = resistant; CPE = carbapenemase-producing *Enterobacterales*.

**Figure S1. Directed Acyclic Graph (DAG)**

ALT: Directed acyclic graph (DAG) illustrating the hypothesized causal relationships between carbapenemase-producing *Enterobacterales* (CPE) bloodstream infection diagnosis and 30-day all-cause mortality. The node “CPE BSI diagnosis” represents exposure of interest. The mediators in the causal pathway were ICU admission at onset, Pitt bacteremia score, and adequate targeted therapy. Confounders included age, immunosuppression, pneumonia as an infection source, and concordant empirical therapy. The diagram depicts arrows from confounders to both exposure and outcome, from CPE infection to mediators, and finally to mortality. This model guided covariate selection for multivariable regression, and CPE infection, while concordant empirical therapy was not significantly protective after adjustment.

**Figure S2. Forest Plot of Adjusted Hazard Ratios**

ALT: Forest plot displaying adjusted hazard ratios (aHRs) for 30-day all-cause mortality associated with CPE versus non-CPE bloodstream infection. The dashed vertical line at HR = 1.0 indicates the reference (non-CPE) group. Blue points show the main cohort analysis; red points represent the cohort excluding COVID-19 patients; and green points represent the *K. pneumoniae* subgroup. Horizontal bars indicate the 95% confidence intervals on a logarithmic scale.

**Table S1.**

ALT: The Schoenfeld residual test results confirmed that the proportional hazards assumption was met for all variables in the Cox regression, including pathogen type, age, pneumonia source, septic shock, concordant therapy, and immunosuppression.

**Table S2.**

ALT: Table comparing comorbidities, risk factors, and sources of bacteremia between patients with CPE and non-CPE bloodstream infections. Significant differences observed in obesity, COVID-19, catheter use, and pneumonia.

**Table S3.**

ALT: Table comparing clinical characteristics and infection sources among patients with KPC-producing, NDM-producing, and non-CPE *Enterobacterales* bacteremia. ICU admission and pneumonia were more common in KPC and NDM groups.

**Table S4.**

## ALT: Multivariable Cox regression analysis showing predictors of 30-day mortality in *Enterobacterales* bacteremia. KPC-producing pathogens and pneumonia were associated with the highest mortality risk.

**Table S5.**

ALT: Sensitivity analysis excluding COVID-19 patients, showing consistent predictors of 30-day mortality. CPE infection remained independently associated with an increased risk of death (aHR 2.27, 95% CI 1.39–3.71; *P* = 0.001).

**Table S6.**

ALT: Baseline characteristics and adjusted analysis of factors associated with 30-day mortality in *K. pneumoniae* bloodstream infection. CPE infections showed higher mortality than non-CPE infections, largely mediated by pneumonia and septic shock.

**Table S7.**

ALT: Outcomes of CPE bacteremia according to definitive antimicrobial regimens. Colistin-based combination therapy was the most frequently used regimen. Mortality rates remained high across all combinations, reflecting limited treatment options.

**Table S8.**

ALT: Antimicrobial susceptibility profiles of CPE and non-CPE *Enterobacterales* bloodstream isolates. CPE isolates showed universal resistance to carbapenems and β-lactams, while susceptibility was retained for amikacin and partially for tigecycline and ceftazidime/avibactam.
