# Supplementary figures and images for: Thirty-Day Mortality Associated With Carbapenemase-Producing Enterobacterales Bloodstream Infections at a Referral Hospital in Peru, 2020–2023
Source: Open Forum Infect Dis. 2025 Dec 1;12(12):ofaf729. doi: 10.1093/ofid/ofaf729 (PMC12692351; doi:10.1093/ofid/ofaf729)

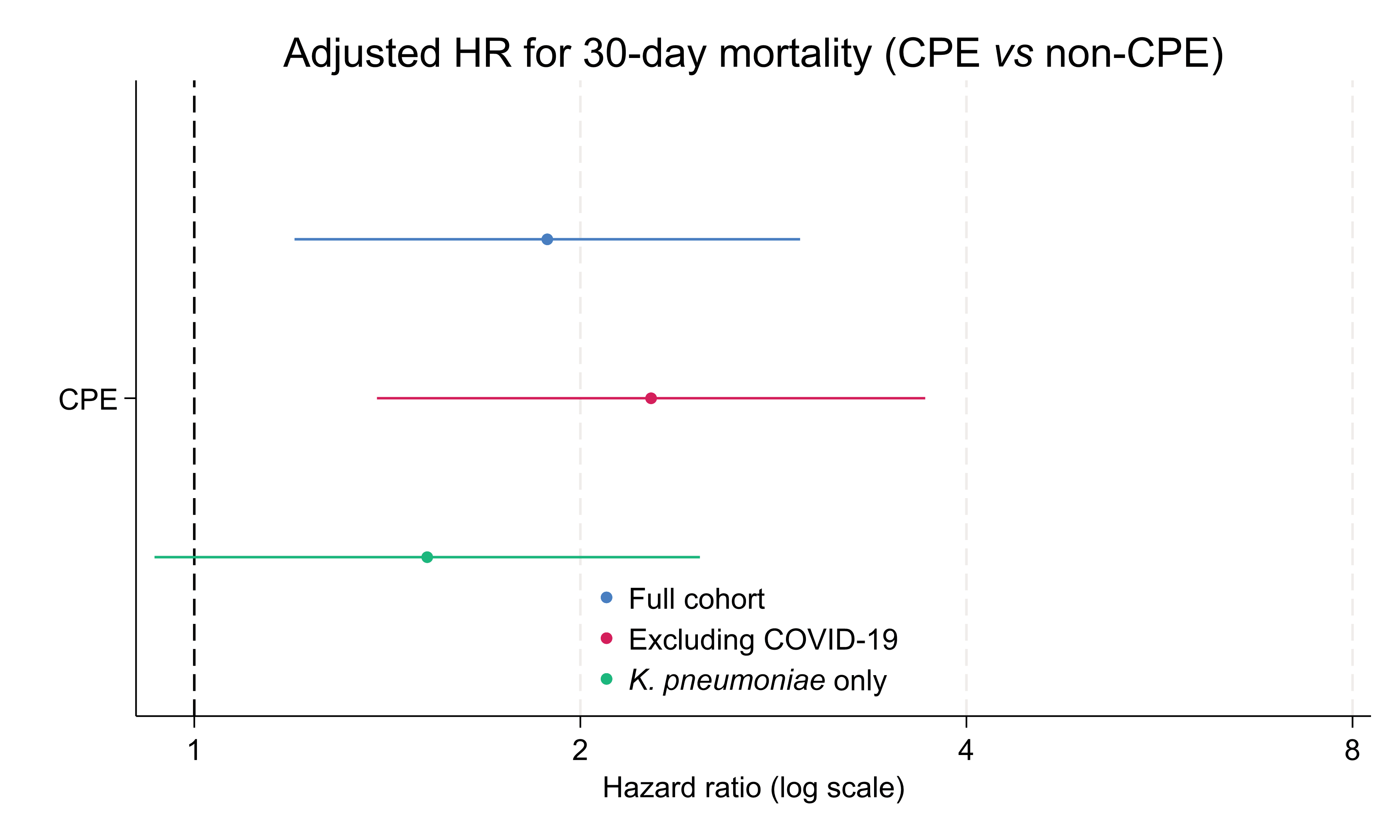

Supplement: ofaf729_Supplementary_Data [file ofaf729_supplementary_data.zip › Figure_S2_ForestPlot.tiff]
